# An open label, dose response study to determine the effect of a dietary supplement on dihydrotestosterone, testosterone and estradiol levels in healthy males

**DOI:** 10.1186/1550-2783-5-12

**Published:** 2008-08-12

**Authors:** Fru Angwafor, Mark L Anderson

**Affiliations:** 1 Yaounde Teaching Hospital, Head of Urological Service Department, University of Yaounde, Cameroon; 2 Director of Research & Development, Triarco Industries, Inc. 400 Hamburg Turnpike, Wayne, NJ, 07418, USA

## Abstract

**Background:**

Maintaining endogenous testosterone (T) levels as men age may slow the symptoms of sarcopenia, andropause and decline in physical performance. Drugs inhibiting the enzyme 5α-reductase (5AR) produce increased blood levels of T and decreased levels of dihydrotestosterone (DHT). However, symptoms of gynecomastia have been reported due to the aromatase (AER) enzyme converting excess T to estradiol (ES). The carotenoid astaxanthin (AX) from *Haematococcus pluvialis*, Saw Palmetto berry lipid extract (SPLE) from *Serenoa repens *and the precise combination of these dietary supplements, Alphastat^® ^(Mytosterone(™)), have been reported to have inhibitory effects on both 5AR and AER in-vitro. Concomitant regulation of both enzymes in-vivo would cause DHT and ES blood levels to decrease and T levels to increase. The purpose of this clinical study was to determine if patented Alphastat^® ^(Mytosterone(™)) could produce these effects in a dose dependent manner.

**Methods:**

To investigate this clinically, 42 healthy males ages 37 to 70 years were divided into two groups of twenty-one and dosed with either 800 mg/day or 2000 mg/day of Alphastat^® ^(Mytosterone(™)) for fourteen days. Blood samples were collected on days 0, 3, 7 and 14 and assayed for T, DHT and ES. Body weight and blood pressure data were collected prior to blood collection. One-way, repeated measures analysis of variance (ANOVA-RM) was performed at a significance level of alpha = 0.05 to determine differences from baseline within each group. Two-way analysis of variance (ANOVA-2) was performed after baseline subtraction, at a significance level of alpha = 0.05 to determine differences between dose groups. Results are expressed as means ± SEM.

**Results:**

ANOVA-RM showed significant within group increases in serum total T and significant decreases in serum DHT from baseline in both dose groups at a significance level of alpha = 0.05. Significant decreases in serum ES are reported for the 2000 mg/day dose group and not the 800 mg/day dose group. Significant within group effects were confirmed using ANOVA-2 analyses after baseline subtraction. ANOVA-2 analyses also showed no significant difference between dose groups with regard to the increase of T or the decrease of DHT. It did show a significant dose dependant decrease in serum ES levels.

**Conclusion:**

Both dose groups showed significant (p = 0.05) increases in T and decreases in DHT within three days of treatment with Alphastat^® ^(Mytosterone(™)). Between group statistical analysis showed no significant (p = 0.05) difference, indicating the effect was not dose dependent and that 800 mg/per day is equally effective as 2000 mg/day for increasing T and lowering DHT. Blood levels of ES however, decreased significantly (p = 0.05) in the 2000 mg/day dose group but not in the 800 mg/day dose group indicating a dose dependant decrease in E levels.

## Background

The process of male aging is associated with a slow progressive decrease in serum testosterone (T) levels as a result of decreased production. It has been designated as T deficiency syndrome (TDS) by the International Society of Andrology (ISA), International Society for the Study of the Aging Male (ISSAM) and European Association of Urology (EAU) [1-3]. Its clinical features can be described as a combination of sarcopenia and andropause leading to a decline in physical performance [4]. Maintaining endogenous T levels as the male ages may slow the symptoms of sarcopenia characterized by muscle erosion, loss of muscle strength and bone mineral density. It may also play a key role in eliminating the symptoms of andropause, characterized by sexual dysfunction, lack of energy, increased cognitive impairment and decreased general well being [5-7]. Avoiding or slowing these symptoms is important for staying healthy and competitive as life expectancy increases. Many of them can be alleviated by T replacement therapy either transdermally or by injection however, they are only available by prescription because of the potential risks of this treatment [5]. T is converted to either dihydrotestosterone (DHT) by the enzyme 5α-reductase (5AR) or estradiol (ES) by the aromatase (AER) enzyme. Recent studies have shown that DHT administered to orchidectomized mice resulted in increased high-density lipoprotein cholesterol and triglyceride levels [8]. DHT is also implicated in the etiology of benign prostate hyperplasia (BPH) which is regarded as a global health problem in men over 50 years of age [9]. Elevated levels of DHT, prostate specific antigen (PSA) and symptoms of BPH are commonly treated with a 5AR inhibitor which reduces the amount of T converted to DHT [10]. However, 5AR inhibitors produce increased blood levels of ES and have been reported to cause symptoms of gynecomastia [11]. Therefore, a dietary supplement that increases endogenous levels of T while decreasing levels of DHT and ES may be very useful for maintaining physical performance and alleviating the conditions of andropause and sarcopenia while decreasing the risk of BPH.

Recent in-vitro studies report that the carotenoid Astaxanthin (AX) from *Haematococcus pluvialis *is a more potent inhibitor of 5AR than the Saw Palmetto berry lipid extract (SPLE) from *Serenoa repens*. When AX is combined with SPLE in specific amounts as (Mytosterone(™)), it showed a greater inhibition of 5AR than SPLE alone [12]. Further, in-vitro assays show that AX inhibits the AER enzyme which converts T to ES [13]. Therefore, we hypothesize that a precise combination of AX and SPLE, Alphastat^® ^(Mytosterone(™)), would regulate the inhibitory activity of both 5AR and AR enzymes in-vivo. Concomitant regulation of both enzymes would cause DHT and ES blood levels to decrease and T levels to increase in a dose dependant manner.

To investigate this, two groups of 21 healthy male participants between the ages of 40 and 70 years were dosed with either 800 or 2000 mg of Alphastat^® ^(Mytosterone(™)) per day over a two week period. Serum levels of T, DHT and ES were analyzed to determine changes in hormone levels.

## Methods

### Study design

This design was a single centre, prospective, open-label, dose comparison clinical study. Two groups of healthy, male subjects were dosed with either 800 mg/day or 2000 mg/day of a precise combination of AX and SPLE (Alphastat^® ^(Mytosterone(™)), Triarco Industries, Wayne, NJ) for 14 consecutive days. Subjects were assigned to dose groups in an alternating manner based on arrival time. Serum levels of T, DHT and ES were used to investigate differences between the two dose groups. Since the purpose of the study was to compare the differences between a high dose and a low dose from baseline values, a control group was not used. Blood samples were drawn at approximately the same time of day to minimize any diurnal variations in hormone levels.

### Subjects

Two groups of twenty-one healthy, males ages 37–70 years volunteered for this study. The ages of the 800 mg/day dose group ranged from 37 years to 67 years with an average of 55.6 years and body weight ranged from 68.4 kg to 102.5 kg with an average of 80.3 kg. The ages of the 2000 mg/day dose group ranged from 53 years to 70 years with an average of 61 years and body weight ranged from 67.9 kg to 95.3 kg with an average of 84.8 kg. Inclusion criteria required subjects to have a PSA lower than 10.1 ng/ml, and pass a compliance screening test and a health screen for malaria, diabetes and ailments mentioned in the exclusion criteria. Exclusion criteria included any cases of moderately severe co-morbid disease including cardiac, pulmonary, renal, hepatic, or active cancer, BPH with complications, abnormal digital rectal examination (DRE), active prostate cancer or history of acute bacterial prostatitis. Also excluded were individuals taking, or who had taken within the past 30 days, any dietary supplements containing saw palmetto or astaxanthin. There were no food or exercise requirements or restrictions. Participants were advised and encouraged to maintain their current dietary intake and exercise habits throughout the study period to prevent changes in either from interfering with the study results. This study was approved by the University of Yaoundé I Teaching Hospital Review Board and the Cameroon Research Ethics Committee. All subjects enrolled in the study completed a written Institutional Review Board (IRB)-approved informed consent and were examined by an urologist at the hospital.

### Dose protocol

The low dose group (n = 21) ingested two 400 mg capsules per day, one in the morning and one in the evening. The high dose group (n = 21) ingested five 400 mg capsules per day, 2 in the morning, 1 in the afternoon and 2 in the evening. All capsules were taken with 8–12 oz of water.

### Diet

There were no diet protocol or diet log requirements however, participants were advised and encouraged to maintain their current dietary intake habits throughout the study period in order to prevent changes in dietary intake confounding the study results.

### Sample collection

Blood samples were collected between 8 am and 10:30 AM on the sampling days. Sample collection was conducted at the hospital using red capped Vacutainer^® ^tubes and centrifuged to produce serum. All tubes were coded, refrigerated and sent to the Laboratory of Nutrition and Nutritional Biochemistry for analyses.

### Serum analyses

Serum levels of T, DHT, and ES were determined using separate commercial ELISA test kits (Alpha Diagnostics, San Antonio, USA). All reference standards, controls and serum samples were dispensed at room temperature. All test kits have been designed and tested for human serum samples.

### DHT determination in serum

A 50 μL aliquot of each standard, control and sample were dispensed into separate antibody coated wells of the strip plate. DHT – Horseradish peroxidase (HRP) conjugate solution (100 μL) was then dispensed into each well, the plates covered and incubated at room temperature for 60 minutes with gentle shaking. Following incubation the plate was aspirated and washed three times with approximately 300 μL diluted wash buffer. HRP-substrate solution, Tetramethylbenzidine (TMB) (150 μL) was then added into each well, the plates covered and incubated at room temperature for 10 minutes with gentle shaking (approximately 200 rpm) to develop a blue color. Stopping solution (50 μL) was then added into each well and the plates mixed gently as the blue color turned yellow. The concentration of each well was determined by absorbance measured at 450 nm. Within run variations in control values of greater than +/- 20% required reanalysis. The specificity of DHT ELISA kit was determined by measuring interference from high concentrations of the following: DHT 100%; T, 8.7%; 5-beta-DHT, 2%; Androstenedione, 0.2%; Dehydroepiandrosterone sulfate, 17-beta-estradiol, Estriol, Estrone, Progesterone, 17-OH-Progesterone, Cortical pregnenolone < 0.01%.

### ES determination in serum

A 50 μL aliquot of each standard, control and sample were dispensed into separate anti-Mouse IgG coated wells of the strip plate. ES-HRP conjugate solution (100 μL) was then added into each well. The plate was covered and incubated at room temperature for 60 minutes with gentle shaking. Following incubation the plate was aspirated and washed three times with approximately 250 μL diluted wash buffer. HRP substrate, TMB solution (150 μL) was added to each well, the plates covered and incubated at room temperature for 10 minutes with gentle shaking to develop a blue color. Stopping solution (50 μL) was then added into each well and the plates mixed gently as the blue color turned yellow. The concentration of each well was determined by absorbance measured at 450 nm. Within run variations in control values of greater than +/- 20% required reanalysis. The ES antibody used in this kit is very sensitive and specific. The following compounds were tested for cross reactivity of the assay: ES (100%), Estriol and Estrone (1%), Progesterone, and Cortisol (0.1%).

### Total T determination in serum

A 10 μL aliquot of each standard, control and sample were dispensed into the appropriate wells and rabbit polyclonal antibody solution into all wells of the strip plate. Enzyme conjugate solution (50 μL) was then added to each well. The plate was covered and incubated at room temperature for 60 minutes with gentle shaking. Following incubation the plate was aspirated and washed 3 times with approximately 250 μL diluted wash buffer. HRP substrate Solution A (100 μL) and HRP substrate Solution B (100 μL) was added to each well. The plate covered and incubated at room temperature for 30 minutes with gentle shaking. Stopping solution (50 μL) was then added into each well and the plates mixed gently as the blue color turned yellow. The concentration of each well was determined by absorbance measured at 450 nm. Within run variations in control values of greater than +/- 20% required reanalysis. The rabbit polyclonal antibody used in this kit is very sensitive and specific for T. The following compounds were tested for cross-reactivity of the assay: T (100%), 5-a-dihydrostestosterone (9.6%), Androstenedione (1.7%), 11-oxystestosterone (1.5%), Epiandrosterone (0.06%). The following compounds had negligible cross-reactivity: 5-beta-DHT, 5-a-androstan-3-a, 17b-estradiol, 17b-Diol, 5-a-androstan-3, 17 dione, Androsterone, Cortisol, Dehydroepiandrosterone, Estriol, Estrone, Progesterone, Corticosterone, Danazol, 11-b-hydroxytestosterone.

### Statistical analysis

All significance and power testing on results was done at a level of alpha = 0.05. Within group analyses was performed on T, DHT and ES levels between baseline and each time point, within each dose group using one-way repeated measures analysis of variance (ANOVA-RM). Two-way analysis of variance (ANOVA-2) was performed on T, DHT and ES levels between each dose group after baseline subtraction. Results are expressed as means ± SEM. Statistics were performed using a commercially available software program (Origin^® ^for Windows, version 8.0).

## Results

### Body weight and tolerance

The mean baseline body weight was 80.3 kg in the 800 mg/day dose group and 84.8 kg in the 2000 mg/day dose group. There was no significant change in mean body weight over the 14 day treatment period in either dose group. Both doses of Alphastat^® ^(Mytosterone(™)) were well tolerated and no adverse events reported.

### Blood pressure

The mean baseline systolic blood pressure (SBP) was 142 mmHg in the 800 mg/day dose group and 137 mmHg in the 2000 mg/day dose group. No significant (p = 0.05) changes in mean SBP from baseline values were reported in the 800 mg/day dose group. In the 2000 mg/day group SBP was reported to be significantly (p < 0.05) below baseline values on day 3, day 7 and day 14. The mean baseline Diastolic blood pressure (DBP) was 71.5 mmHg in the 800 mg/day dose group and 66.9 mmHg in the 2000 mg/day dose group. DBP was reported to be significantly (p < 0.05) below baseline values on day 7 and day 14 in both the 800 mg/day dose group and 2000 mg/day dose group.

### Serum T levels

**Figure 1:**
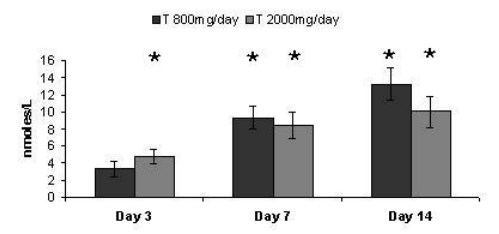
**Serum Total Testosterone**. Effect of Alphastat^® ^(Mytosterone(™)) on total testosterone levels (nmol/L). Values are means (+/- SEM), * indicates significant difference from baseline value (p ≤ 0.05).

The mean baseline level of serum total T in the 800 mg/day dose group of 21.64 nmol/L was significantly (p = 0.05) different than the baseline level of 26.26 nmol/L in the 2000 mg/day dose group. ANOVA-RM of the 800 mg/day dose group showed the mean level of T was significantly (p < 0.05) greater than the mean baseline level at day 7 and day 14. ANOVA-RM of the 2000 mg/day dose group showed the mean level of T was significantly (p < 0.05) greater than the mean baseline level at day 3, day 7 and day 14. ANOVA-2 comparison, after baseline subtraction, between the 800 mg/day dose group and the 2000 mg/day dose group showed the interaction between time points to be significant (p = 0.05) reaching a statistical significance of 1.0. Comparison of means between dose groups showed no significant (p = 0.05) interaction reaching a statistical power of 0.51 (Figure 1).

### Serum DHT levels

**Figure 2:**
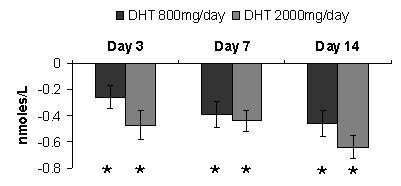
**Serum Dihydrotestosterone**. Effect of Alphastat^® ^(Mytosterone(™)) on serum DHT levels (nmol/L). Values are means (+/- SEM), *indicates significant difference from baseline value (p ≤ 0.05).

The mean baseline level of serum DHT in the 800 mg/day dose group of 2.79 nmol/L was significantly (p = 0.05) different than the baseline level of 2.34 nmol/L in the 2000 mg/day dose group. ANOVA-RM of the 800 mg/day dose group showed the mean level of DHT was significantly (p < 0.05) less than the mean baseline level at day 3, day 7 and day 14. ANOVA-RM of the 2000 mg/day dose group showed the mean level of DHT was significantly less (p < 0.05) than the mean baseline level at day 3, day 7 and day 14. ANOVA-2 comparison, after baseline subtraction, between the 800 mg/day dose group and the 2000 mg/day dose group showed interaction between time points to be significant (p = 0.05) reaching a statistical significance of 1.0. Comparison of means between dose groups showed no significant (p = 0.05) interaction reaching a statistical power of 0.46 (Figure 2).

### Serum ES levels

**Figure 3:**
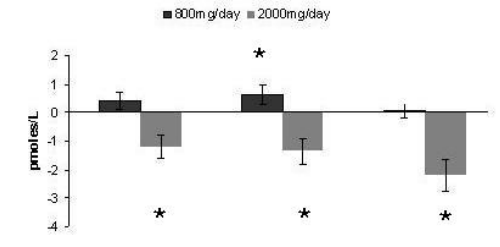
**Serum Estradiol**. Effect of Alphastat^® ^(Mytosterone(™)) on serum estradiol levels (pmol/L). Values are means (+/- SEM), *indicates significant difference from baseline value (p ≤ 0.05).

The mean baseline level of serum ES in the 800 mg/day dose group of 21.49 pmol/L was not significantly (p = 0.05) different than the baseline level of 23.94 pmol/L in the 2000 mg/day dose group. ANOVA-RM of the 800 mg/day dose group showed the mean level of ES was significantly (p < 0.05) less than the mean baseline level at day 7. ANOVA-RM of the 2000 mg/day dose group showed the mean level of ES was significantly less (p < 0.05) than the mean baseline level at day 3, day 7 and day 14. ANOVA-2 comparison, after baseline subtraction, between the 800 mg/day dose group and the 2000 mg/day dose group showed the interaction between time points to be significant (p = 0.05) reaching a statistical significance of 0.73. Interaction between dose groups was also significant (p = 0.05) reaching a statistical power of 1.0 (Figure 3).

## Discussion

ANOVA-RM analyses of the results show significant increases in serum total T and significant decreases in serum DHT in both dose groups as well as significant decreases in serum ES in the 2000 mg/day dose group at a significance level of alpha = 0.05. These significant differences from baseline were confirmed using ANOVA-2 analyses after baseline subtraction. These results of increased T levels with concurrent decreases in DHT and ES support earlier in-vitro mechanism reports that the 5AR and AER enzymes are inhibited by AX and SPLE [12,13]. They also support the hypothesis that a precise combination of AX and SPLE, Alphastat^® ^(Mytosterone(™)), may regulate a concomitant inhibitory activity of both enzymes in-vivo.

The doses used in this study however, did not result in a clear dose dependent effect. ANOVA-2 analyses show no significant difference between dose groups with regard to the increase of T or the decrease of DHT. However, it did show a significant dose dependant decrease in serum ES levels. The lack of a dose response with regard to T and DHT indicates the maximum effect of Alphastat^® ^(Mytosterone(™)) is obtained at the 800 mg/day dose. Further studies using lower doses must be conducted to establish a dose response and maximum effect level regarding T and ES. A dose of 2000 mg/day may be necessary if decreases in ES levels are desired. This effect may have an application in women that are prone to estrogen dependent breast cancer and requires further exploration.

The results also suggest that Alphastat^® ^(Mytosterone(™)) is equally effective in men between the ages of 37 and 70 for increasing endogenous total serum T levels and decreasing serum DHT levels without increasing ES levels. The 800 mg/day dose group ranged 37 – 67 years with a mean of 55.5 years and 24% under the age of 50. The 2000 mg/day dose group ranged 53 – 70 years with a mean of 61 years and no one under the age of 50. Confirmation of this by further studies is important since mean total T levels have been reported to decrease by 30% between the ages of 25 and 75 [14]. The decline in T as a result of male aging is designated as TDS by several international societies and is described as a combination of sarcopenia and andropause [1-3]. Its clinical features are characterized by muscle erosion, loss of muscle strength and bone mineral density as well as increased sexual dysfunction, lack of energy, increased cognitive impairment and decreased general well being [5-7]. Avoiding or slowing these symptoms is important for staying healthy and competitive as life expectancy increases. The results of this study indicate that Alphastat^® ^(Mytosterone(™)) may help maintain or reverse declining T levels in the aging male by maintaining endogenous T levels and without increasing ES levels. It may also be beneficial in males prone to developing symptoms of BPH due to elevated DHT levels. BPH is regarded as a global health problem in men with approximately 50% of men over age 50 reporting symptoms [9,10]. Alphastat^® ^(Mytosterone(™)) may regulate DHT levels without the side effects of increased ES levels reported with the use of prescription drugs that inhibit 5AR.

## Conclusion

The precise combination of AX and SPLE, Alphastat^® ^(Mytosterone(™)), produced significant changes in serum T, DHT and ES levels. A dose of either 800 mg or 2000 mg/day produced significant increases in T and decreases in DHT within three days with no increases in ES. The effect was not dose dependent indicating that the 800 mg/per day dose is as equally effective as 2000 mg/day in the age range of subjects studied. Blood levels of ES also decreased significantly and in a dose dependant manner indicating the 2000 mg/day dose is more effective than the 800 mg/day dose. There were no outward signs of toxicity or adverse reactions. This data provides support for each mechanism of action observed in-vitro and suggests a potential role for its use in aging men experiencing TDS or symptoms of BPH.

## Competing interests

This study was funded by Triarco Industries, Inc. (Wayne, NJ) through the contract research organization (CRO) Gateway Health Alliances, Inc. All research was conducted independently and according to protocol at the Laboratory of Nutrition and Nutritional Biochemistry, University of Yaounde I, Cameroon. All researchers have no financial interests concerning the outcome of this investigation and the results do not constitute an endorsement by the authors and/or their institutions concerning the ingredient tested.

## Authors' contributions

FA assisted in study coordination, supervision, protocol development, data management and statistical analysis. MA assisted in protocol development, clinical supply management and manuscript preparation. All authors read and approved the final manuscript.
